# Acquisition repeatability of MRI radiomics features in the head and neck: a dual-3D-sequence multi-scan study

**DOI:** 10.1186/s42492-022-00106-3

**Published:** 2022-04-01

**Authors:** Cindy Xue, Jing Yuan, Yihang Zhou, Oi Lei Wong, Kin Yin Cheung, Siu Ki Yu

**Affiliations:** 1 Research Department, Hong Kong Sanatorium & Hospital, Hong Kong, China; 2 Medical Physics Department, Hong Kong Sanatorium & Hospital, Hong Kong, China

**Keywords:** Radiomics, Magnetic resonance guided radiotherapy, Head and neck, Repeatability, Intraclass correlation coefficient

## Abstract

Radiomics has increasingly been investigated as a potential biomarker in quantitative imaging to facilitate personalized diagnosis and treatment of head and neck cancer (HNC), a group of malignancies associated with high heterogeneity. However, the feature reliability of radiomics is a major obstacle to its broad validity and generality in application to the highly heterogeneous head and neck (HN) tissues. In particular, feature repeatability of radiomics in magnetic resonance imaging (MRI) acquisition, which is considered a crucial confounding factor of radiomics feature reliability, is still sparsely investigated. This study prospectively investigated the acquisition repeatability of 93 MRI radiomics features in ten HN tissues of 15 healthy volunteers, aiming for potential magnetic resonance-guided radiotherapy (MRgRT) treatment of HNC. Each subject underwent four MRI acquisitions with MRgRT treatment position and immobilization using two pulse sequences of 3D T1-weighed turbo spin-echo and 3D T2-weighed turbo spin-echo on a 1.5 T MRI simulator. The repeatability of radiomics feature acquisition was evaluated in terms of the intraclass correlation coefficient (ICC), whereas within-subject acquisition variability was evaluated in terms of the coefficient of variation (CV). The results showed that MRI radiomics features exhibited heterogeneous acquisition variability and uncertainty dependent on feature types, tissues, and pulse sequences. Only a small fraction of features showed excellent acquisition repeatability (ICC > 0.9) and low within-subject variability. Multiple MRI scans improved the accuracy and confidence of the identification of reliable features concerning MRI acquisition compared to simple test-retest repeated scans. This study contributes to the literature on the reliability of radiomics features with respect to MRI acquisition and the selection of reliable radiomics features for use in modeling in future HNC MRgRT applications.

**Supplementary Information:**

The online version contains supplementary material available at 10.1186/s42492-022-00106-3.

## Introduction

Head and neck cancer (HNC) represents a group of malignancies associated with high heterogeneity, not only in terms of organs and tissues of origin, but also etiological, molecular, and mutational differences [1]. The global incidence of HNC has been continuously rising in recent decades [2]. Treatment options for HNC treatment include surgery, radiation therapy (RT), chemotherapy, targeted therapy, immunotherapy, and combinations of these methods. However, the heterogeneity of HNC partially accounts for the frequency of unsatisfactory treatment outcomes, particularly in advanced stages [3].

Traditionally, magnetic resonance imaging (MRI) has played an important role in the diagnosis, prognosis, and treatment planning of HNC by virtue of its superior soft-tissue image contrast and various functional imaging capabilities [4–7]. In recent years, with the introduction of treatment with MRI-integrated linear accelerator (MR-LINAC) systems to clinical use [8–10], the role of MRI in HNC has considerably extended from conventional diagnosis to image-based guidance of radiation delivery in RT, referred to as MR-guided radiotherapy (MRgRT) [9]. Despite in its infancy, MRgRT has shown promise as an innovative technique for HNC treatment [11–13], allowing for better delineation of target organs and organs at risk (OARs), daily treatment plan adaptation, real-time motion monitoring, gating, and tracking for dose delivery, as well as intra- and inter-fractional treatment response evaluation.

There has been an increased demand for developing biomarkers to facilitate personalized diagnosis and treatment of HNC. Radiomics [14–16] has attracted increasing research interest as a multidimensional data mining technique in medical imaging for the diagnosis and prognosis of HNC in recent years [17–21]. Despite the promising results reported in these studies, the reliability of radiomics features remains a major obstacle to the broad validity and generality of radiomics in routine clinical use [22–24].

Image acquisition is a crucial procedure that substantially influences radiomics feature values for all imaging modalities, in particular for MRI [25–30]. Firstly, the image intensity of normal anatomical MRI is semi-quantitative, being comprehensively influenced by many tissue properties such as relaxation times, proton density, fat-water composition, and susceptibility, without representing an exact physical meaning. Second, different hardware and configurations of MRI scanners from various vendors considerably impact image quality and characteristics and thus radiomics features. Third, the variety of MRI pulse sequences, imaging parameters, and reconstruction algorithms also dramatically influences MR image contrast and quality. Moreover, radiomics features values can also vary with organ motion during acquisition and the administration of contrast agents.

To facilitate radiomics in HNC MRgRT, the acquisition repeatability of MRI radiomics features prior to the use of these features must be investigated directly for diagnosis or prognosis modeling. Of note, MRgRT exhibits some unique characteristics compared to diagnostic MRI. In contrast to diagnostic MRI, in which one (for cross-sectional studies) or two (for longitudinal studies) MRI scans per patient are normally involved, multiple MRI scans are required in MRgRT fractions to obtain the required daily anatomical information for treatment adaptation. MRI acquisition in MRgRT relies heavily on 3D pulse sequences to obtain isotropic voxel sizes and better geometric fidelity than 2D sequences. In particular, 3D T2-weighted (T2W) turbo spin-echo (TSE) is heavily utilized in MRgRT without the need for administration of a contrast agent. In addition, patients are typically scanned with flexible RF coils that are compatible with the immobilized treatment position, rather than diagnostic volumetric coils. Finally, radiomics in MRgRT mainly utilizes within-subject inter-fractional longitudinal radiomics feature variation for response evaluation and treatment adaptation, which is also known as delta-radiomics [31, 32], in contrast to the between-subject radiomics feature difference used in diagnostic radiology to perform lesion differentiation or characterization.

Thus, in this study, several aims were considered in investigating the repeatability of the acquisition of MRI radiomics features for MRgRT applications specifically. They were (1) to identify repeatable MRI features in two pulse sequences of 3D T1-weighed turbo spin-echo (3D-T1-TSE) and 3D T2-weighed turbo spin-echo (3D-T2-TSE), (2) to investigate whether feature acquisition repeatability varies with different HN tissues; (3) to evaluate whether and how a multi-scan study design could impact the determination of repeatable radiomics features compared to the commonly used test-retest (repeated scan) study design, and (4) to establish a benchmark for feature variability in MRI acquisition from normal HN tissues of use in future research on delta-radiomics in MRgRT.

## Methods

This study was approved by the research ethics committee of Hong Kong Sanatorium and Hospital. A total of 15 healthy volunteers (8 men and 7 women with ages ranging from 24 to 40 years) were prospectively recruited for this study. Informed consent was obtained from each subject.

### MRI acquisition

All MRI scans were conducted using a 1.5 T MRI scanner dedicated to radiotherapy simulation (MR-sim) (Magnetom Aera, Siemens Healthineers, Erlangen, Germany). Each volunteer underwent four scans (with an interval of approximately 15 mins between scan) while immobilized with a 5-pin head, neck, and shoulder thermoplastic mask (Orfit Industries, Belgium). For each scan, the volunteers were precisely aligned using a 3-dimensional external laser (DORADOnova MR3T, LAP GmbH Laser Applikationen, Luneburg, Germany) and scanned in the same treatment position on an RT-indexed flat coach top (Diacor, Salt Lake City, Utah, USA). Two flexible 4-channel flexible coils, one 18-channel flexible coil, and a spine coil were used in combination for MRI signal reception. Each scan consisted of a 3D-T1W-TSE sequence followed by a 3D-T2W-TSE sequence. A vendor-provided received B1 field-inhomogeneity correction, i.e., prescan normalization, was conducted to minimize the bias field of the MR images. 3D geometric distortion correction was also enabled on the console for image acquisition. The imaging parameters prescribed for each sequence are listed in Table. 1.

**Table 1:** Imaging parameters of the two 3D pulse sequences

**Scanning Sequence**	3D-T1W-TSE	3D-T2W-TSE
**TR/TE [ms]**	420/7.2	2300/303
**Echo train length**	40	185
**FOV (LR × SI × AP)[mm**^**3**^**]**	470 × 470 × 269	470 × 470 × 269
**Matrix size (LR × SI × AP)**	448 × 448 × 256	448 × 448 × 256
**Voxel size [mm**^**3**^**]**	1.05 × 1.05 × 1.05	1.05 × 1.05 × 1.05
**Acceleration factor (GRAPPA)**	3	3
**Partial Fourier factor**	6/8	6/8
**Pixel Bandwidth [Hz/pixel]**	657	620
**Scan duration (mm:ss)**	05:01	05:24

*LR* Left/right, *SI* Superior/inferior, *AP* Anterior/posterior, *GRAPPA* Generalized autocalibrating partial parallel acquisition

### Image registration and volumes-of-interest drawing

The digital imaging and communications in medicine format MR images were imported to the 3D Slicer v 4.10.2 [33] for registration and volume of interest (VOI) drawing. The first-scan 3D-T1W-TSE MRI set was used as the positional reference for image registration. Other images were rigidly registered to the reference 3D-T1W-TSE images to compensate for the residual positional shifts, although these shifts have been reported to be very small (approximately several millimeters) owing to the immobilization of the subject by the thermoplastic mask [34, 35].

**Fig. 1:**
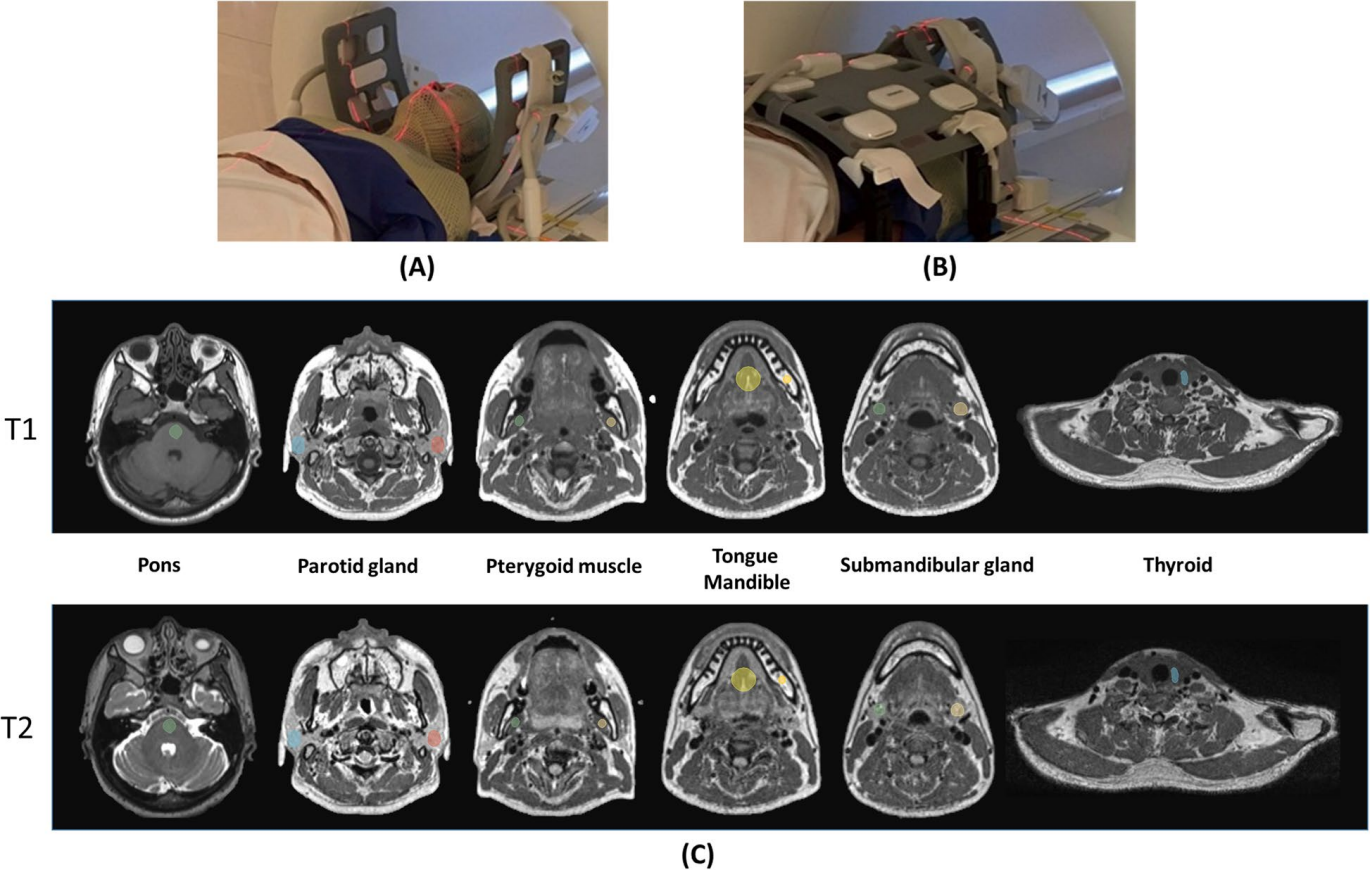
Subject positional verification setup and the acquired 3D TSE MR images. (**a**) A subject immobilized using thermoplastic mask aligned with the external laser system; (**b**) a subject setup and the coil setting on the MR-sim; (**c**) all ten VOIs on MRI image

Ten spherical or ellipsoidal VOIs of pons (2075.34 ± 675.17 mm^3^), left (L) and right (R) parotid glands (L: 5693.71 ± 2614.65 mm^3^, R: 5336.53 ± 2497.18 mm^3^), mandible (1008.58 ± 526.79 mm^3^), tongue (4077.71 ± 1030.59 mm^3^), L/R pterygoid muscle (L: 2455.70 ± 992.56 mm^3^, R: 2290.83 ± 927.36 mm^3^), thyroid (550.40 ± 243.95 mm^3^), and L/R submandibular gland (L: 2241.13 ± 966.31 mm^3^, R: 2255.08 ± 880.35 mm^3^) were manually drawn by an MRI physicist on the first-scan (reference) 3D-T1W-TSE images and validated by a second MRI physicist. Then, all VOIs were propagated to other registered image sets and visually checked by both MRI physicists to ensure the tissue coherence of the propagated VOIs. A typical MRI scan setup and VOIs overlaid on the 3D-T1W-TSE and 3D-T2W-TSE images are shown in Fig. 1.

### Radiomics feature extraction and calculation

3D radiomics features were calculated using PyRadiomics v.2.2.0 [36]. Ninety-three first-order and texture radiomics features in five categories of gray-level co-occurrence matrix (GLCM), gray-level dependence matrix (GLDM), gray-level run length matrix (GLRLM), gray-level size zone matrix (GLSZM), and neighboring gray-tone difference matrix (NGTDM), mostly compliant with Image Biomarker Standardization Initiative (IBSI) standards [37, 38] (first-order, *n* = 18; texture_GLCM, *n* = 24; texture_GLDM, *n* = 14; texture_GLRLM, *n* = 16; texture_GLSZM, *n* = 16; texture_NGTDM, *n* = 5) were extracted from the original MRI images. Shape radiomics features were not included in the analysis mainly because they were theoretically independent of MRI acquisition and constant VOIs were applied for all MRI datasets. For the mathematical definition of each radiomics feature, the reader is referred to the PyRadiomics documentation (https://pyradiomics.readthedocs.io/en/latest/features.html).

The default bin size of 25 in the software was used to perform image intensity discretization. No scaling of the image voxel size was applied due to the isotropic voxel size of the acquired images. Image intensity normalization was not performed because the images were acquired using a single MRI scanner with fixed imaging parameters. No image denoising, filtering, or other post-processing was conducted prior to radiomics feature calculation to minimize their influence on feature values [39]. Default configuration settings were applied in PyRadiomics for feature calculation unless otherwise specified.

### Data analysis

#### Inter-scan acquisition repeatability of radiomics features

The intraclass correlation coefficient (ICC) (2-way mixed effects, absolute agreement, single rater) calculated based on all four MRI scans was used to assess the acquisition repeatability of radiomics features. The feature repeatability was classified as excellent (ICC > 0.9), good (0.9 > ICC > 0.75), moderate (0.75 > ICC > 0.5), and poor (ICC < 0.5) when the calculated ICC and its 95%CI were both within the thresholds according to Koo and Li [40]. If the 95%CI of the calculated ICC was located across two or more ranges, the corresponding feature was classified as the lowest repeatability class. Based on the ICC classification, the radiomics features showing excellent acquisition repeatability were identified for each VOI and each sequence. Then, features universally showing excellent acquisition repeatability in all VOIs for both pulse sequences were identified.

To assess whether multi-scan substantially affected the calculated ICC values and feature repeatability determination, the ICCs were also calculated based on the first two and the first three MRI scans, and compared to the corresponding ICCs based on all four MRI scans.

The intra-subject radiomics feature variability due to multi-scan acquisition was quantified in terms of the coefficient of variation (CV_intra-subject_), defined as the ratio of the standard deviation (SD) to the mean of the radiomics feature values calculated from four MRI scans. Similarly, inter-subject radiomics feature variability was quantified by CV_inter-subject_, defined as the ratio of the SD to the mean of radiomics feature values across all subjects.

#### Statistical analysis

Descriptive statistics were represented in the form of mean ± SD. The Mann-Whitney U-test was conducted to compare the (4-scan derived) ICC values between T1 and T2 pulse sequences. The Kruskal-Wallis test was used to compare ICCs derived from two, three, and four MRI scans for each sequence. The Mann-Whitney U-test and Wilcoxon signed-rank test were also conducted to compare the CVs for different VOIs and feature categories in each pulse sequence. A *p*-value smaller than 0.05 indicated statistical significance. All statistical tests were conducted using RStudio 2021.09.0 Build 351 (RStudio PBC, Boston, MA, USA).

## Results

### Inter-scan acquisition repeatability of radiomics features

**Fig. 2:**
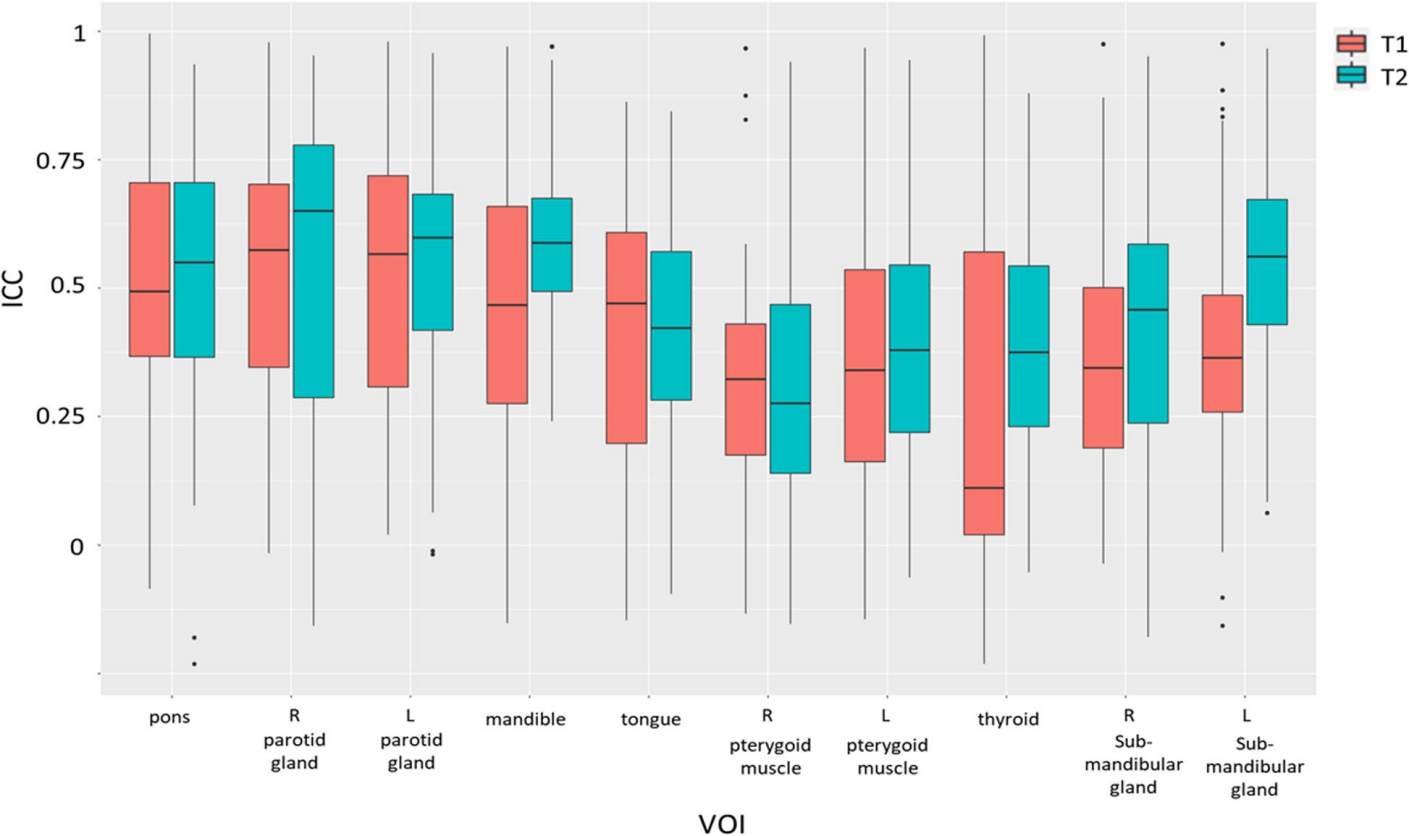
Boxplots of ICC values in different tissue VOIs for both pulse sequences

The repeatability of radiomics feature acquisition assessed by ICC varied with feature categories, VOIs, and pulse sequences. Figure 2 shows boxplots of ICC in different VOIs for both pulse sequences. As shown in Fig. 2, the ICC values were significantly different (ANOVA, *p* < 0.001) between different VOIs for both pulse sequences. In general, ICCs associated with 3D-T1W-TSE (0.418 ± 0.270) were significantly lower (*p* < 0.001) than those associated with 3D-T2W-TSE (0.473 ± 0.249), implying that better feature acquisition repeatability could be obtained with the 3D-T2W-TSE sequence. For 3D-T1W-TSE, the VOI of the R parotid gland showed the highest ICCs (0.539 ± 0.250), whereas the VOI of the thyroid showed the lowest (0.283 ± 0.355). In comparison, for 3D-T2W-TSE, the VOI of the R parotid gland also showed the highest ICCs (0.548 ± 0.285), whereas the VOI of the R pterygoid muscle showed the lowest ICCs (0.316 ± 0.258). The ICCs of the paired VOIs of L/R parotid gland, L/R pterygoid muscle, and L/R submandibular gland did not differ significantly (all *p* > 0.05) for both pulse sequences.

**Fig. 3:**
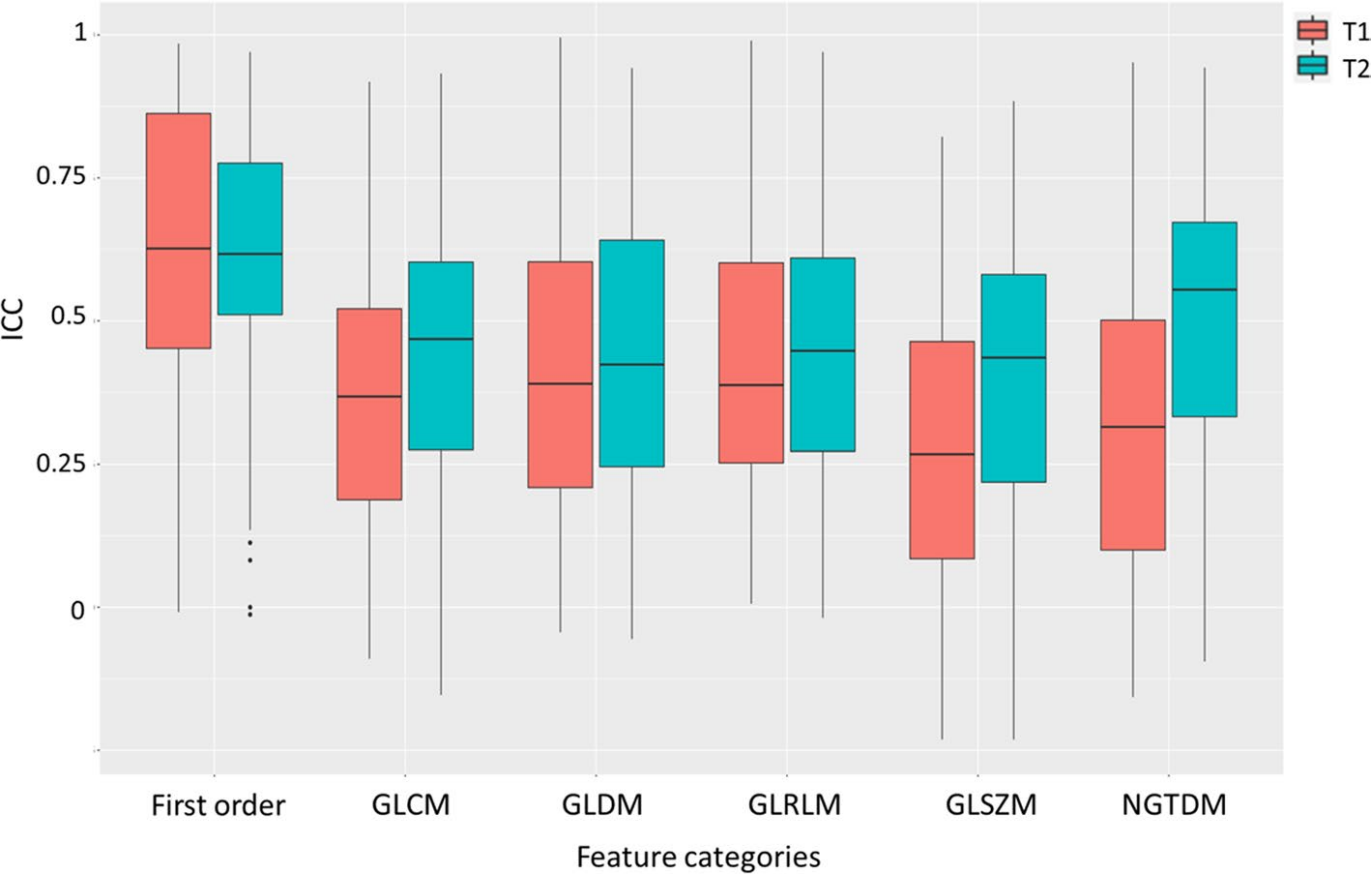
Boxplots of ICC values for different radiomics feature categories in all VOIs for both pulse sequences

Figure 3 shows boxplots of ICCs of different radiomics feature categories for all VOIs for both pulse sequences. The ICCs of first-order radiomics features were significantly higher (*p* < 0.001) than those of texture radiomics features for both sequences. The GLSZM features exhibited the lowest ICCs (T1: 0.279 ± 0.242; T2: 0.387 ± 0.262) in all feature categories for both sequences.

**Fig. 4:**
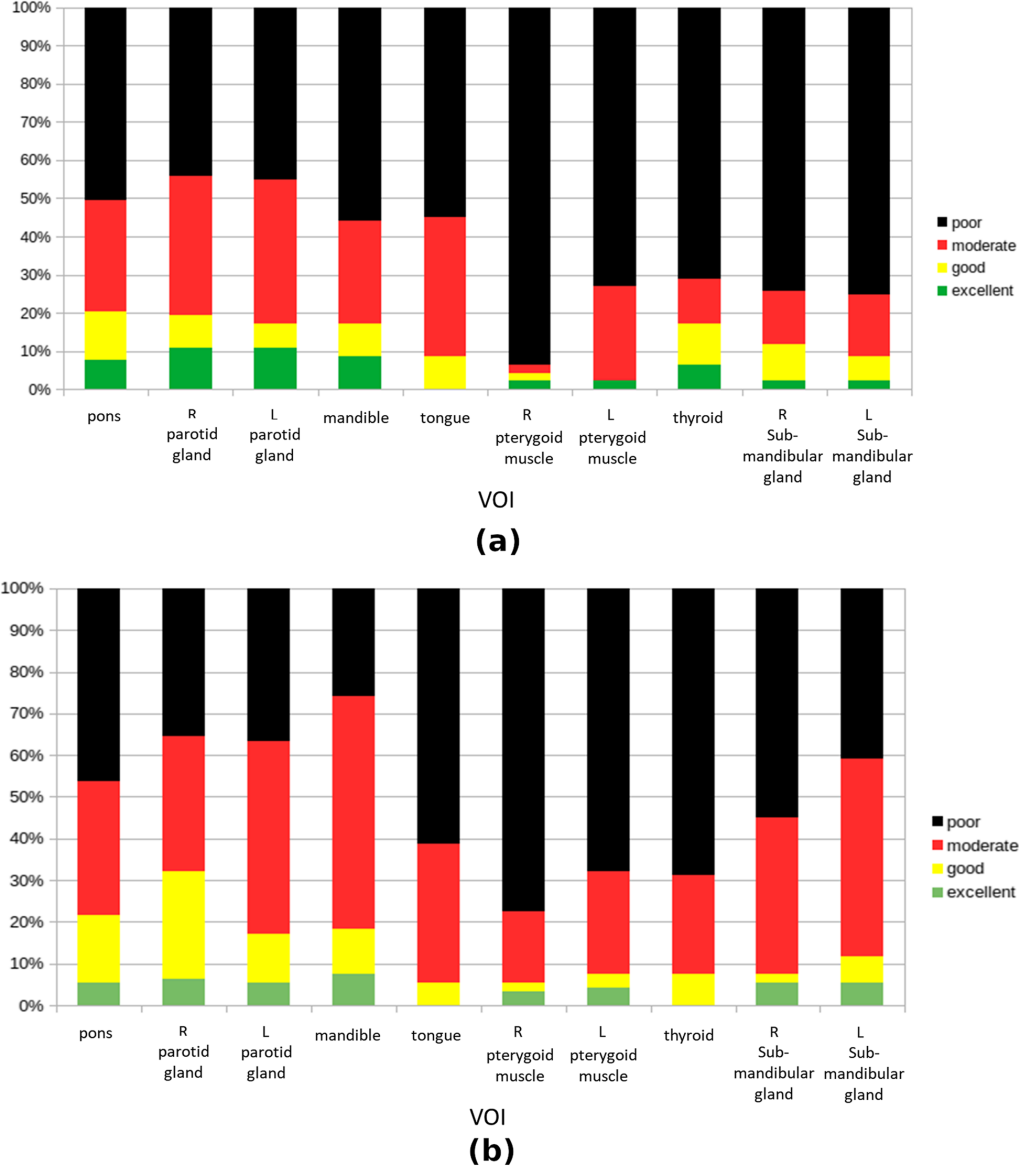
Percentages of excellent, good, moderate, and poor ICCs of radiomics features in different tissue VOIs for (**a**) 3D-T1W-TSE and (**b**) 3D-T2W-TSE pulse sequences

Figure 4 illustrates the percentages of radiomics features showing excellent, good, moderate, and poor ICCs in different VOIs for the 3D-T1W-TSE and 3D-T2W-TSE sequences. There were only (5.27% ± 4.00%) and (4.41% ± 2.66%) radiomics features that showed excellent repeatability for 3D-T1W-TSE and 3D-T2W-TSE, respectively, in different VOIs. For both sequences, the VOI of the R parotid gland exhibited the largest number of excellent repeatability features (T1W: 10.75%, 10/93; T2W: 7.53%, 7/93), while tongue had no excellent repeatability feature at all.

**Fig. 5:**
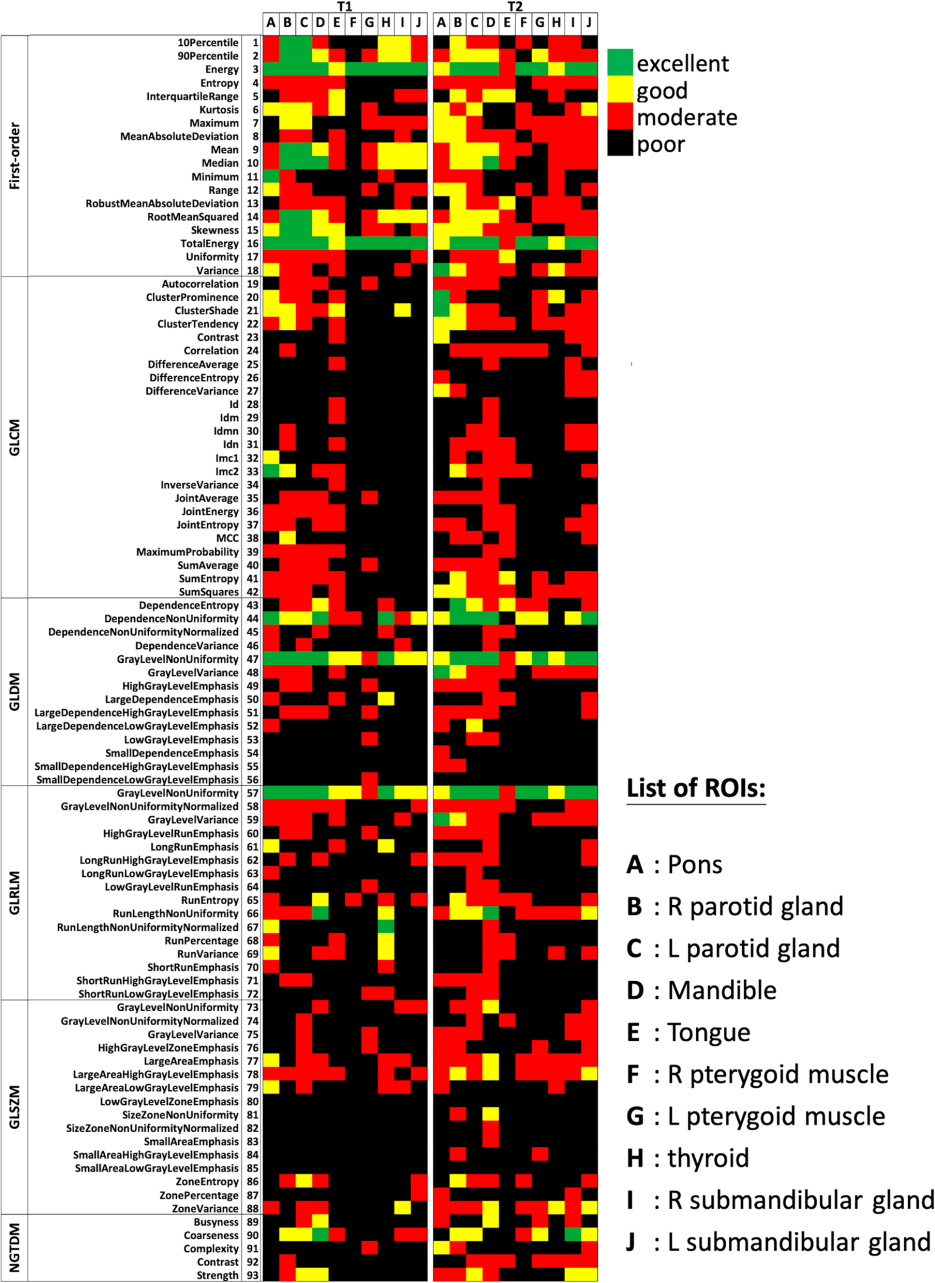
Acquisition repeatability of radiomics features for each VOI and pulse sequence. Excellent (ICC > 0.9), good (0.9 > ICC > 0.75), moderate (0.75 > ICC > 0.5), and poor (ICC < 0.5) repeatability was marked by green, yellow, red, and black blocks respectively

Figure 5 shows the radiomics features with their acquisition repeatability classifications for each VOI and each sequence. Only four features showed mostly good or excellent ICCs in all VOIs (except for the tongue), without significant differences in the ICC between the two sequences. They were firstorder_Energy, firstorder_TotalEnergy, GLDM_GrayLevelNonUniformity, and GLRLM_GrayLevelNonUniformity. These four features were highly repeatable and robust to image acquisition using the two 3D pulse sequences.

For both 3D-T1W-TSE and 3D-T2W-TSE, the ICCs calculated from two, three, and four repeated MRI scans were not significantly different from each other (all *p* > 0.05). However, the 95%CIs associated with the ICCs calculated from fewer repeated MRI scans widened significantly. Boxplots of ICC values based on two or three MRI scans in different tissue VOIs for both pulse sequences are shown in Supplementary Fig. 1. The ICCs calculated from two or three MRI scans moderately affected the repeatability of radiomics feature acquisition. Fewer radiomics features showed excellent or good repeatability, but more features showed poor repeatability due to the much wider 95%CI of the ICC (indicating a larger uncertainty of the calculated ICC value) calculated from fewer scans. The percentages of radiomics features showing excellent, good, moderate, and poor ICCs based on two and three MRI scans in different VOIs for two sequences were demonstrated by the bar plots given in Supplementary Fig. 2.

**Fig. 6:**
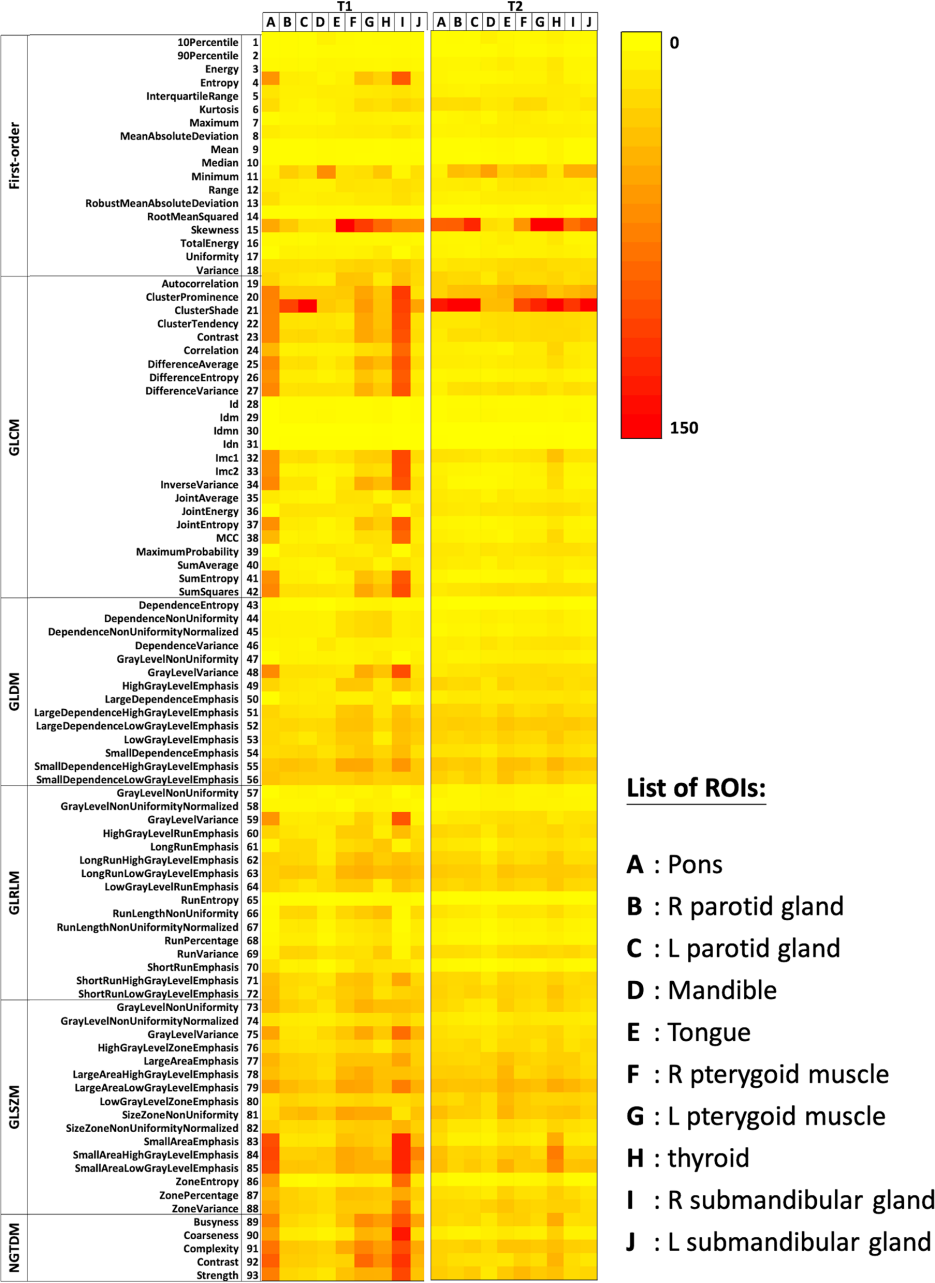
The heatmaps depicting CV_intra-subject_ values of all radiomics features in different VOIs for 3D-T1W-TSE and 3D-T2W-TSE pulse sequences

**Fig. 7:**
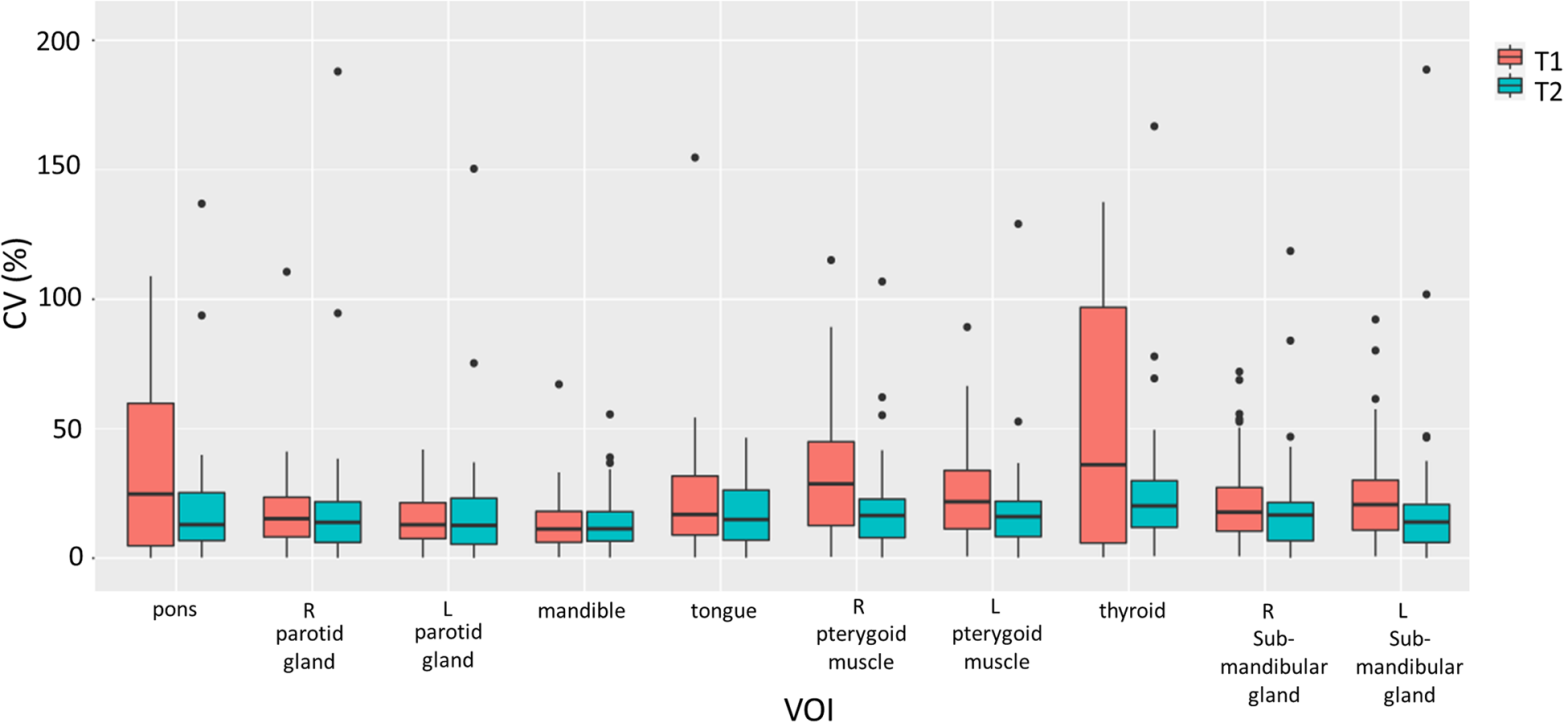
Boxplots of the feature CV_intra-subject_ values in different tissue VOIs for both pulse sequences

The heatmaps shown in Fig. 6 depict the CV_intra-subject_ of all radiomics features in different VOIs for both pulse sequences. The CV_intra-subject_ of features were 24.57% ± 25.36% and 18.06% ± 23.34% for 3D-T1W-TSE and 3D-T2W-TSE, respectively, exhibiting pronounced variability in the values of radiomics features in multi-scan acquisitions, with a significant difference (*p* < 0.001) between the two sequences. In comparison, the CV_intra-subject_ was significantly lower than the CV_inter-subject_ (3D-T1W-TSE: 49.70% ± 72.97%; 3D-T2W-TSE: 51.22% ± 142.09%) (*p* < 0.05). The results showed a significant difference in CV_intra-subject_ between the first-order and texture features for both 3D-T1W-TSE and 3D-T2W-TSE (*p* < 0.05). The boxplots in Fig. 7 illustrate the within-subject feature CV_intra-subject_ with regard to the tissue VOIs. There was a significant difference in CV_intra-subject_ (*p* < 0.05) between tissues. The VOI of the mandible showed the smallest CV_intra-subject_ (T1: 13.44% ± 9.97%; T2: 12.80% ± 9.26%), whereas the VOI of the thyroid showed the largest CV (T1: 47.17% ± 43.37%; T2: 25.36% ± 30.79%) in both sequences.

## Discussion

This study prospectively investigated the multi-scan acquisition repeatability and variability of IBSI-compliant MRI radiomics features for two pulse sequences of 3D-T1W-TSE and 3D-T2W-TSE in a cohort of healthy volunteers, to support potential applications of MRI radiomics in HNC MRgRT. To the best of our knowledge, the present work is the first to investigate the repeatability of radiomics features for the acquisition of MRI data on the HN specifically.

In recent years, an increasing number of studies have reported promising preliminary results on the use of radiomics for the diagnosis and prognosis of HNC [17–21]. However, the broad reproducibility, validity, and generality of radiomics remain open to question or challenge owing to the wide variety of confounding factors that could substantially impact every procedure of the complicated radiomics workflow and lead to uncertainty, instability, or unreliability of the results of radiomics analysis [22–24]. To date, the accumulated evidence remains insufficient to justify the deployment of radiomics in routine clinical practice.

This study specifically addressed an important source of MRI radiomics feature unreliability due to image acquisition, and identified repeatable features from two 3D sequences, which are expected to be helpful in the selection of reliable radiomics features and thus support modeling in future HNC MRgRT applications. The CVs presented in the results should also be useful to establish a reference benchmark of MRI radiomics feature variability for acquisition from normal HN tissues for future research on MRgRT delta-radiomics.

Some notable findings were observed that deserve discussion. First, only a very small proportion of the investigated features could achieve excellent multi-scan repeatability measured by ICC, which is generally consistent with the results of many previous MRI radiomics studies, although in different anatomies [25, 26, 30, 41–43]. However, the proportion of features showing excellent ICC obtained in this study was even smaller than that reported in prior works. In addition to high heterogeneity of the multiple HN tissues, this finding could also be attributed to the ICC calculation based on the four-scan MRI data and more stringent ICC classification by its 95%CI. First-order features were more repeatable than texture features, which also accords with previous studies [26, 44–47]. Second, regarding sequence dependence, the mean feature ICC was better with 3D-T2W-TSE than with 3D-T1W-TSE for most tissue VOIs (Fig. 2) and for most feature categories (Fig. 3). Fewer features showed poor ICC (< 0.5) in 3D-T2W-TSE than in 3D-T1W-TSE (Fig. 4). These results might be partially explained by the fact that T2W MR Images typically exhibit a wider voxel intensity range than T1W MR Images. That is, T1W MR Images have more uniform intensities in many tissues, which causes many texture features to show small inter-subject differences in their values, thus leading to low ICC values. However, the number of features showing excellent ICC was not necessarily larger in 3D-T2W-TSE for the different VOIs (Fig. 4). Third, feature repeatability was also found to be tissue-dependent. Different tissues could exhibit substantially different feature ICCs owing to their intrinsic properties, heterogeneities, and thus different image representations. This finding indicates that different features might be chosen for the construction of tissue-specific radiomics models for clinical use. Next, multiple MRI scans provided additional information on feature repeatability compared to the simple test-retest repeated scans. Fewer scans were found to have a wider 95%CI and thus a larger uncertainty of the calculated ICC values, which might lead to overestimation of feature repeatability if based on the ICC value alone. However, by referring to the 95%CI of ICC for feature repeatability classification, the highly repeatable features for MRI acquisition could be more accurately identified via multi-scan than by dual-scan. In addition, multi-scan enabled the calculation of feature CV_intra-subject_ to assess within-subject feature value variability, whereas dual-scan could only evaluate the feature value difference between two scans. This within-subject feature variability is of particular importance for individualized radiomics analysis in MRgRT studies, in which longitudinal MRI datasets obtained from multiple treatment fractions of each subject are used. It is crucial for MRgRT to accurately differentiate the true variations in the value of radiomics features due to response to irradiation from variation or uncertainty due to image acquisition. Otherwise, radiomics analysis could result in false positives discovery. The CV_intra-subject_ results obtained in this study revealed that many features were subjected to pronounced value variations in multiple MRI scans. The CV_intra-subject_s were much larger than the previously reported values in a longitudinal phantom study using the same model MRI scanner [48]. This is not surprising because in vivo tissues exhibit much higher heterogeneity and are subjected to much more intra- and inter-scan tissue property change, positional variation, deformation, and motion. Thus, it is vital to carefully select repeatable acquisition features from each patient for reliable delta-radiomics analysis. However, the CV_intra-subject_ was still smaller than the CV_inter-subject_, which indicated that although acquisition-induced feature variability can impact inter-subject radiomics analysis for diagnosis purpose, its impact might not be as great as in within-subject longitudinal radiomics analysis.

This study involves several strengths and limitations. In addition to its prospective nature, this is the first study on MRI radiomics feature acquisition repeatability study in the HN dedicated to MRgRT applications, as noted above. Dedicated 3D pulse sequences for MRgRT were used, and multiple MRI scans were conducted under MRgRT treatment positioning (e.g., 3D laser alignment, flat couch, mask immobilization, etc.) and setting (RF coil selection and coil setting with dedicated holders and bridges). The use of mask immobilization maximized tissue coherence in the VOIs, alleviating the influence of image registration on feature quantification [26]. The multiple-scan study design not only reflected the pattern of MRI use in clinical MRgRT practice but also extended the capability to calculate within-subject feature CV and increased the ICC calculation confidence for more reliable feature selection. The adoption of IBSI-compliant features increased the transparency of the feature calculation. Confounding factors other than image acquisition were excluded as much as possible in the study design and data analysis to minimize their influence on feature values. As such, the results may be considered to faithfully reveal the feature repeatability and variability purely with respect to the acquisition. Paired tissue analysis for calibration was helpful in ensuring the validity and rigor of the quantification results.

However, this study does involve some limitations. This pilot study was mainly limited to the inclusion of only healthy volunteers and a small sample size. Malignant HN tumors might exhibit substantially different between-subject radiomics feature heterogeneities as well as their within-subject measurement uncertainties compared to normal HN tissues, but they could not be revealed because the study was conducted on healthy volunteers. The logistical difficulty and ethical concerns involved in conducting such a multi-scan study on real HNC patients should be recognized. However, despite the absence of malignant tumors in healthy volunteers, it is expected that this pilot study may still be useful for future clinical applications because radiomics can also be used in a variety of OARs, which are thought to be normal tissues but are inevitably irradiated, to assess their toxicities or the quality-of-life of HNC patients treated with MRgRT. The small sample size limited the statistical power of the tests. Although this study was designed with a focus on MRgRT applications, all MRI scans were acquired using an MRI simulator instead of an actual MR-LINAC. Considering the difference in the configurations of MRI hardware and implementations of 3D pulse sequence on a 1.5 T MR-LINAC from those of a 1.5 T MRI simulator from a different vendor, radiomics features and their repeatability/variability characteristics obtained on a 1.5 T MR-LINAC might be considerably different from those obtained in this study. Meanwhile, the approximately 1 mm isotropic spatial resolution obtained in this study was higher than that normally used for daily MRI acquisition in MRgRT. Although it is helpful for better tissue delineation and registration, this high resolution might also influence feature repeatability and variability. This study specifically addressed an important source of MRI radiomics feature unreliability due to multiple acquisitions, but did not assess the influence of many other confounding factors on feature values, such as image reconstruction, segmentation, and other image post-processing methods [49]. Although image acquisition has been found to be more impactful on feature reliability than other sources in MRI radiomics [24], it is equally important to investigate the impact of other factors on the reliability of radiomics features. Meanwhile, only a small subset of first-order and texture original radiomics features was included for analysis in this study among the thousands of radiomics features in the original and transformed images proposed for radiomics modeling in the medical literature. In particular, shape features indicating the geometric characteristics of various tissues (such as size or volume) are conventionally used as imaging markers in cancer staging [50] and treatment response evaluation [51], but were not included in the present work. Although features in the transformed domains might provide more candidates for radiomics modeling and different information on tissue properties, it has been found that image transformation does not necessarily improve radiomics features reliability [24]. Therefore, the absence of transformed features in this study may not seriously compromise the validity and interpretation of the results.

## Conclusion

This prospective study has rigorously investigated the multi-scan acquisition repeatability and variability of MRI radiomics features for 3D-T1W-TSE and 3D-T2W-TSE sequences in normal HN tissues of a cohort of fifteen healthy volunteers using a 1.5 T MRI simulator with MRgRT treatment positioning and coil setting, focusing on providing a benchmark for future MRgRT radiomics studies in HNC. Radiomics features repeatable to each sequence were identified, as measured by ICC. Within-subject variability in multiple MRI acquisitions was quantified. These results are expected to advance the understanding of the reliability of radiomics features with respect to MRI acquisition and the reliable radiomics features selection for modeling in HNC MRgRT applications.

## Supplementary Information

**Additional file 1.**

## Data Availability

The datasets generated and/or analyzed during the current study are not publicly available because the subjects did not provide written consent for their data to be publicly shared.

## Abbreviations

HNC: Head and neck cancer
HN: Head and neck
RT: Radiation therapy
MRI: Magnetic resonance imaging
MR-LINAC: MRI-integrated linear accelerator
MRgRT: Magnetic resonance guided radiotherapy
OARs: Organs at risk
T2W: T2-weighted
TSE: Turbo spin-echo
VOI: Volume of interest
LR: Left/right
SI: Superior/inferior
AP: Anterior/posterior
GRAPPA: Generalized autocalibrating partial parallel acquisition
IBSI: Image Biomarker Standardization Initiative
GLCM: Gray-level co-occurrence matrix
GLDM: Gray-level dependence matrix
GLRLM: Gray-level run length matrix
GLSZM: Gray-level size zone matrix
NGTDM: Neighboring gray-tone difference matrix
ICC: Intraclass correlation coefficient
CV: Coefficient of variation
SD: Standard deviation
ANOVA: Analysis of variance
