# Supplementary material for: Acquisition repeatability of MRI radiomics features in the head and neck: a dual-3D-sequence multi-scan study
Source: Vis Comput Ind Biomed Art. 2022 Apr 1;5:10. doi: 10.1186/s42492-022-00106-3 (PMC8971276; doi:10.1186/s42492-022-00106-3)
Supplement: Supplementary file 1 — Additional file 1. [file 42492_2022_106_MOESM1_ESM.docx]

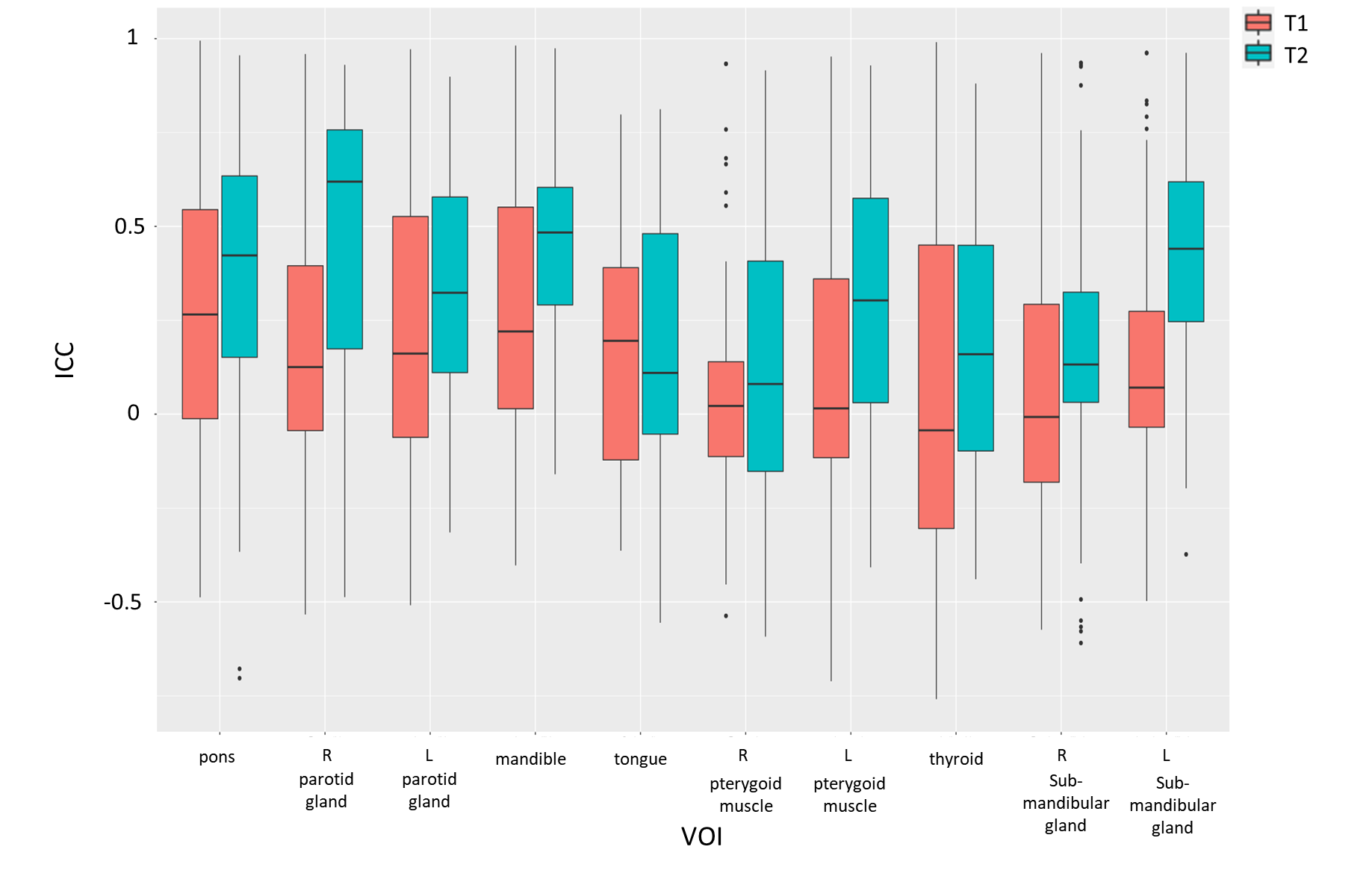
(a)


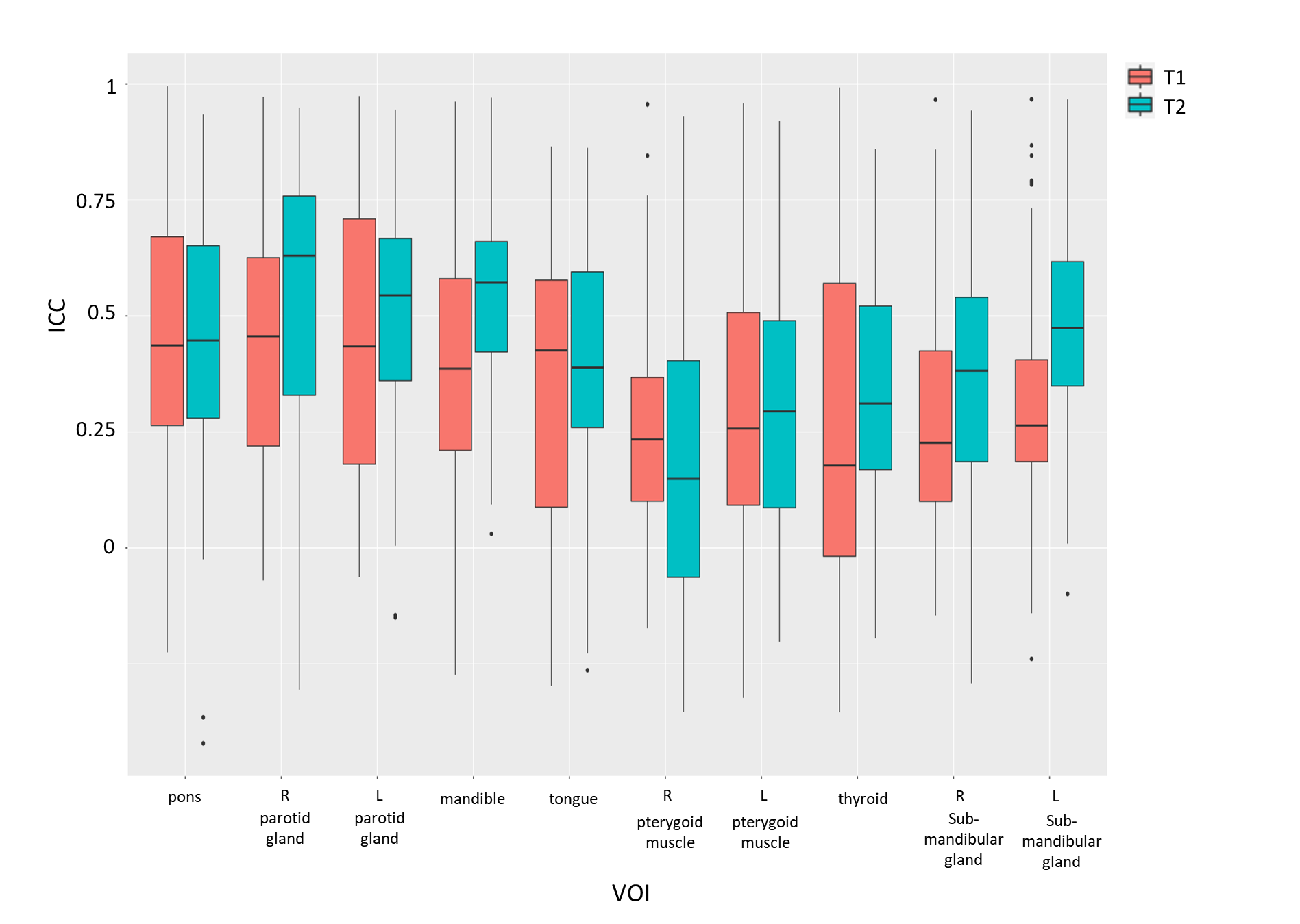


(b)

Supplementary Figure 1. The boxplots of ICC values based on (a) two or (b) three MRI scans in different tissue VOIs for both pulse sequences

| **3D-T1W-TSE** | **3D-T2W-TSE** |
| --- | --- |
| 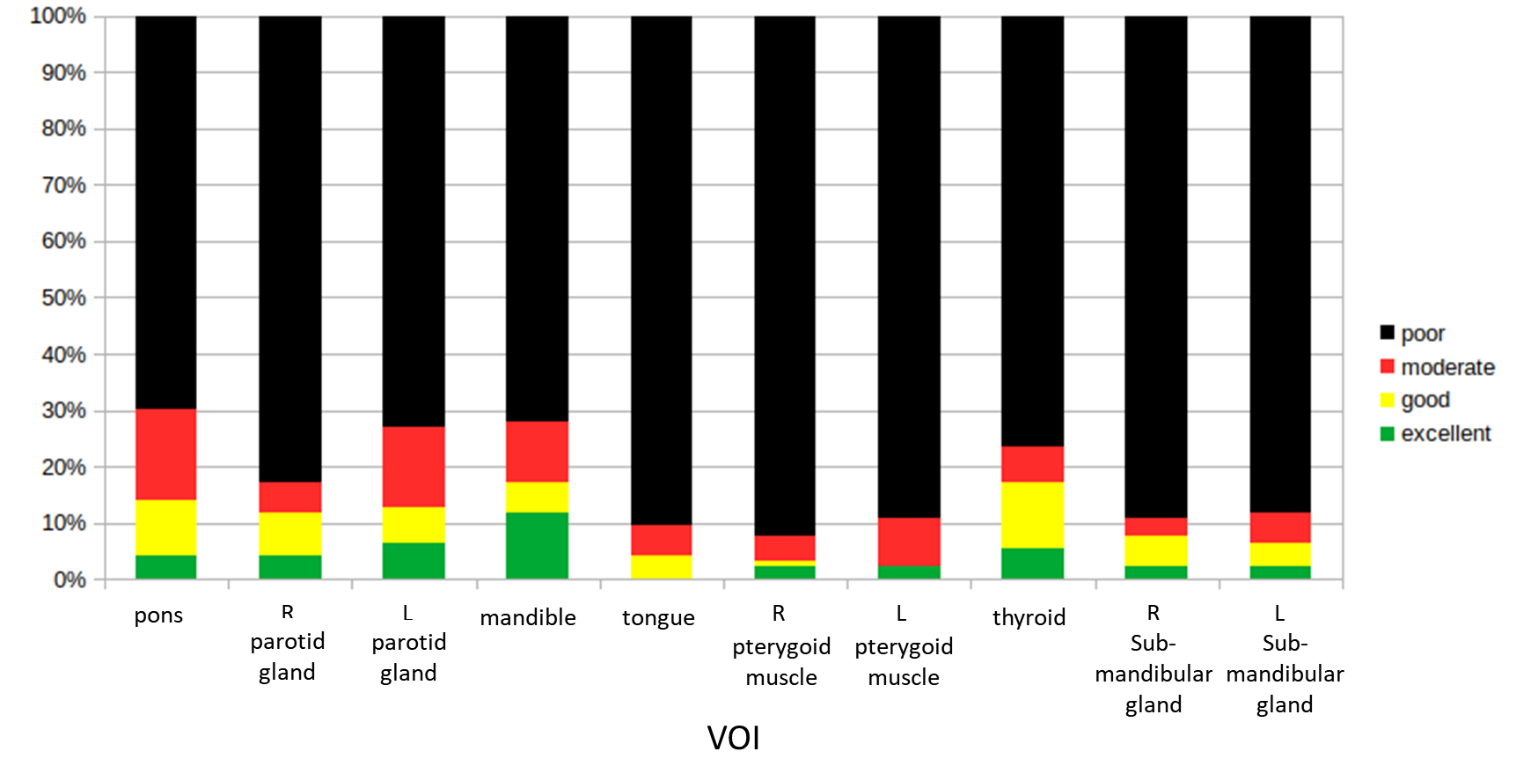  **(a)** | 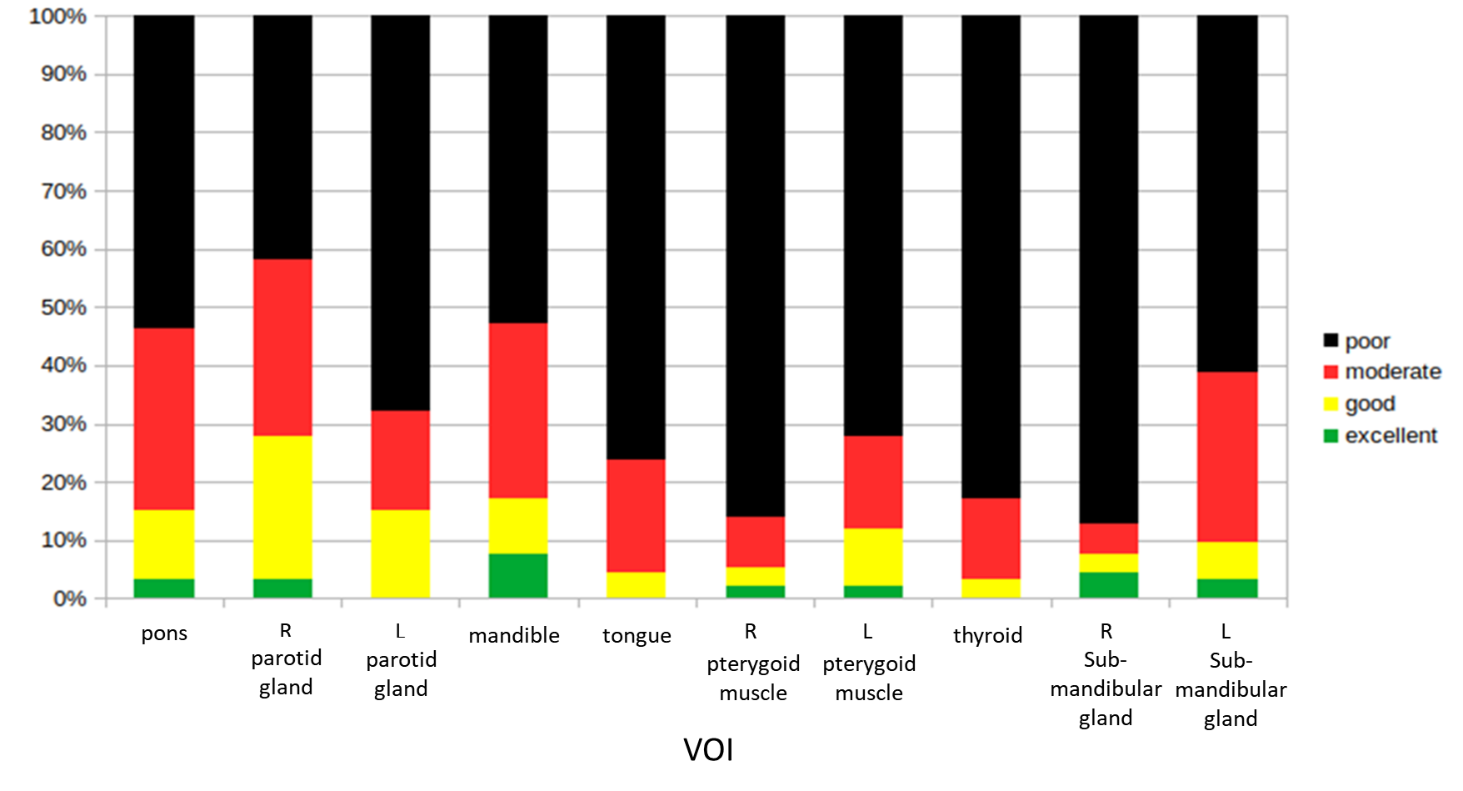  **(b)** |
| 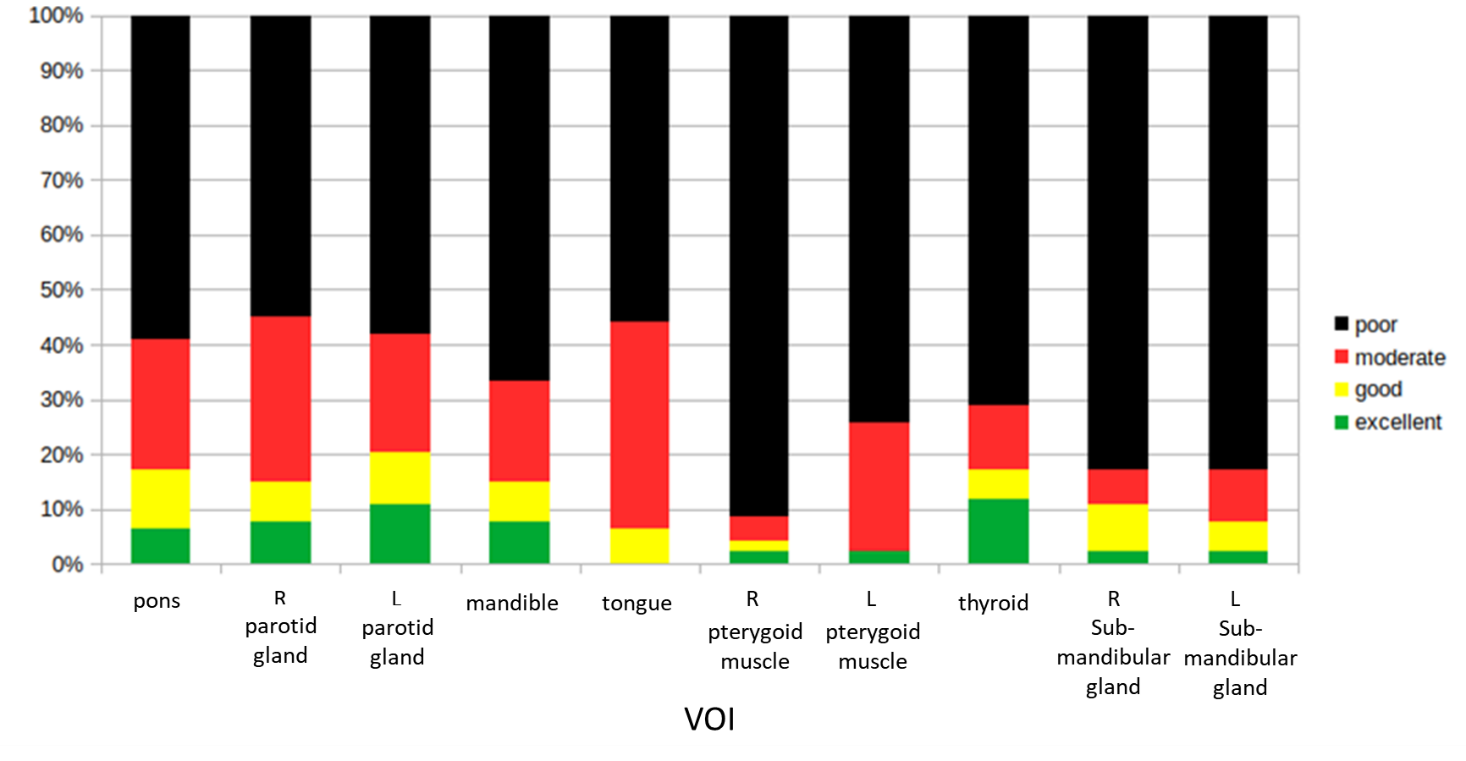  **(c)** | 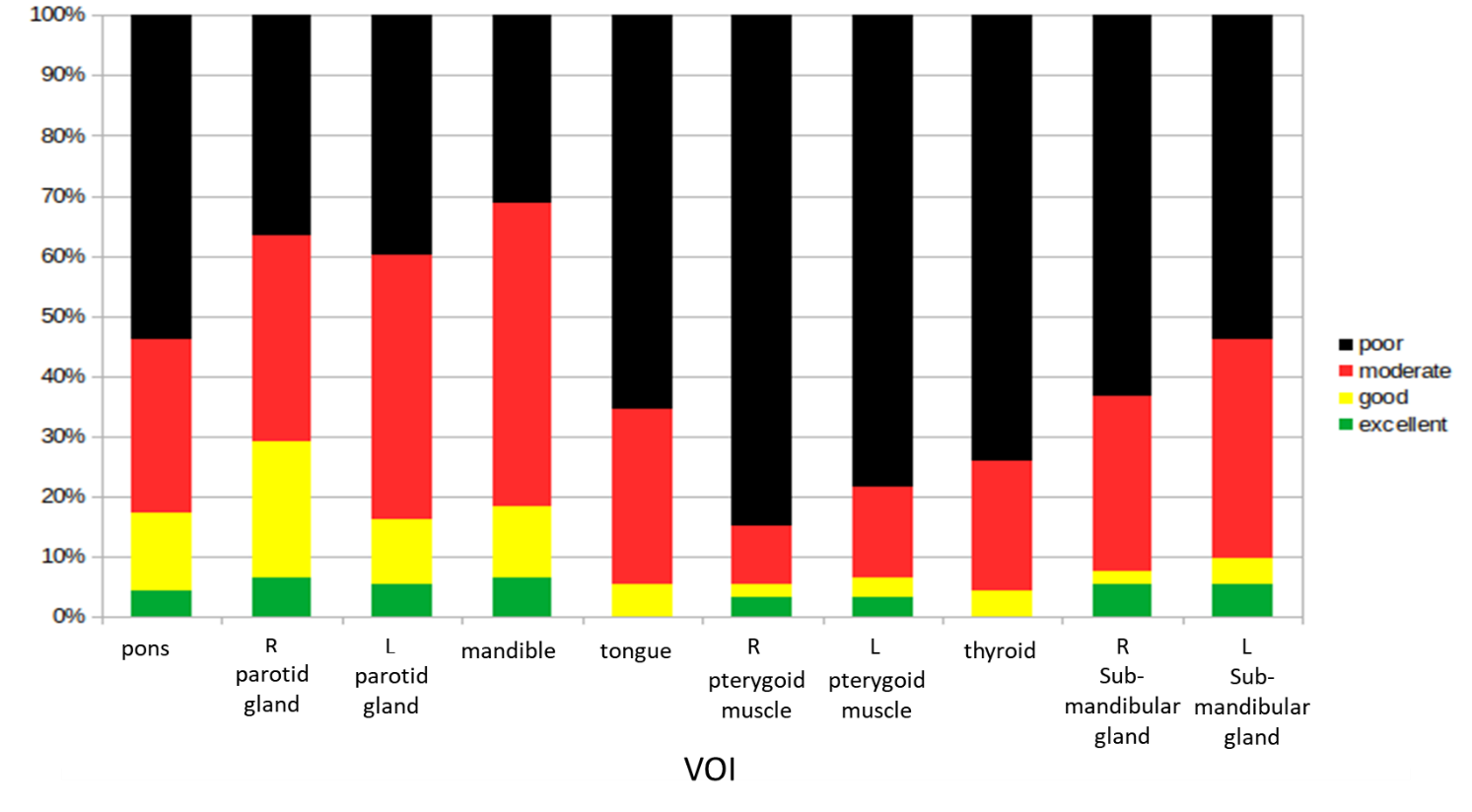  **(d)** |

Supplementary Figure 2. The percentages of excellent, good, moderate, and poor ICCs of radiomics features based on (a-b) two and (c-d) three MRI scans in different tissue volumes-of-interest (VOIs) for (a, c) 3D-T1W-TSE pulse sequence and (b, d) 3D-T2W-TSE pulse sequence.


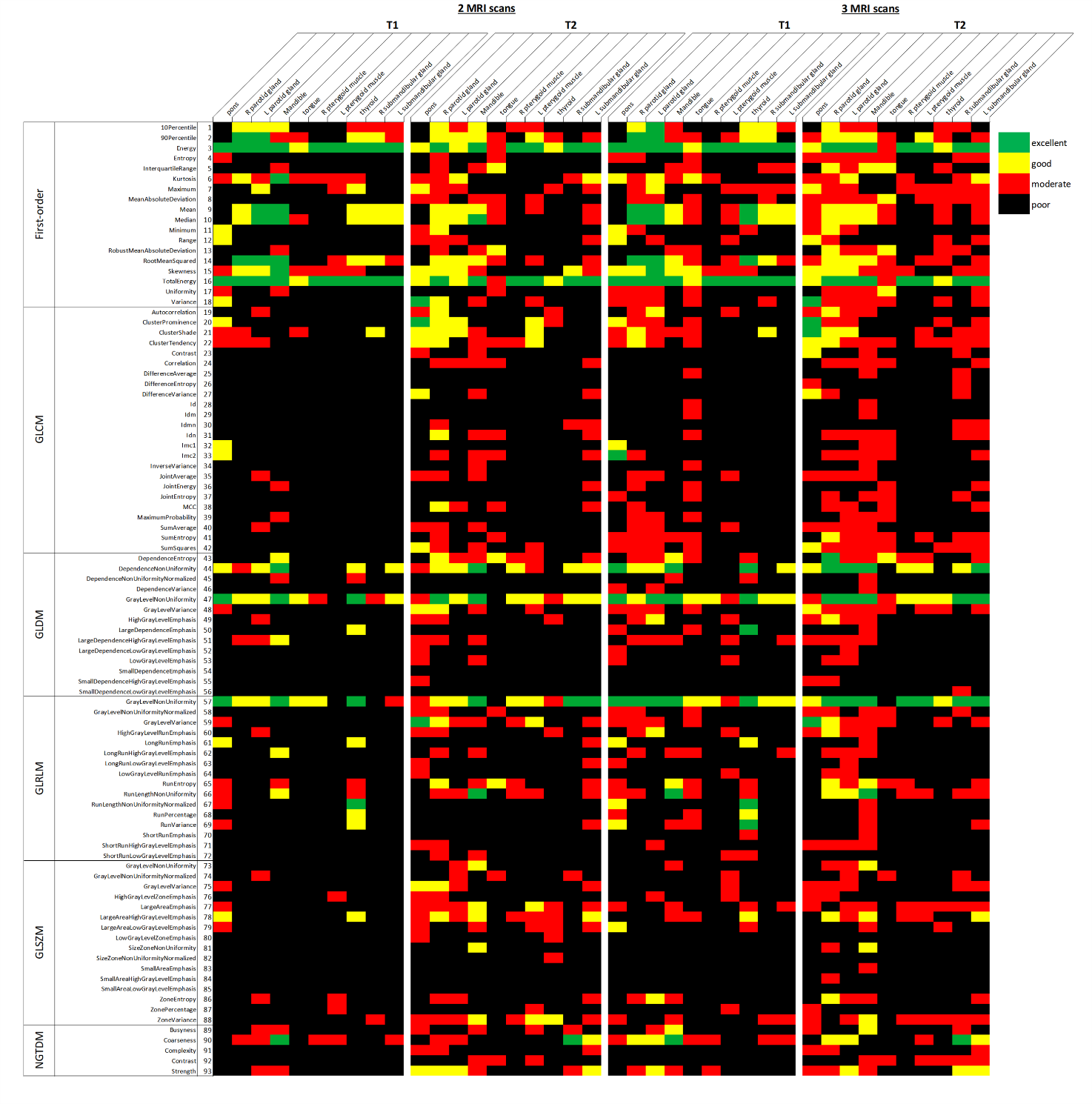


Supplementary Figure 3. The acquisition repeatability of radiomic features for each VOI and pulse sequence based on two and three MRI scans. Excellent (ICC>0.9), good (0.9>ICC>0.75), moderate (0.75>ICC>0.5), and poor (ICC<0.5) repeatability was labeled by green, yellow, red, and black blocks respectively.
